# Supplementary material for: Saffron extract attenuates neuroinflammation in rmTBI mouse model by suppressing NLRP3 inflammasome activation via SIRT1
Source: PLoS One. 2021 Sep 10;16(9):e0257211. doi: 10.1371/journal.pone.0257211 (PMC8432768; doi:10.1371/journal.pone.0257211)
Supplement: S1 Raw images — (PDF) [file pone.0257211.s001.pdf]

Any lane with X symbol is not represented in the figure submitted within the manuscript, we aimed to choose 4 consecutive bands that represent the 4 different experimental groups

GAPDH Original Blot Image

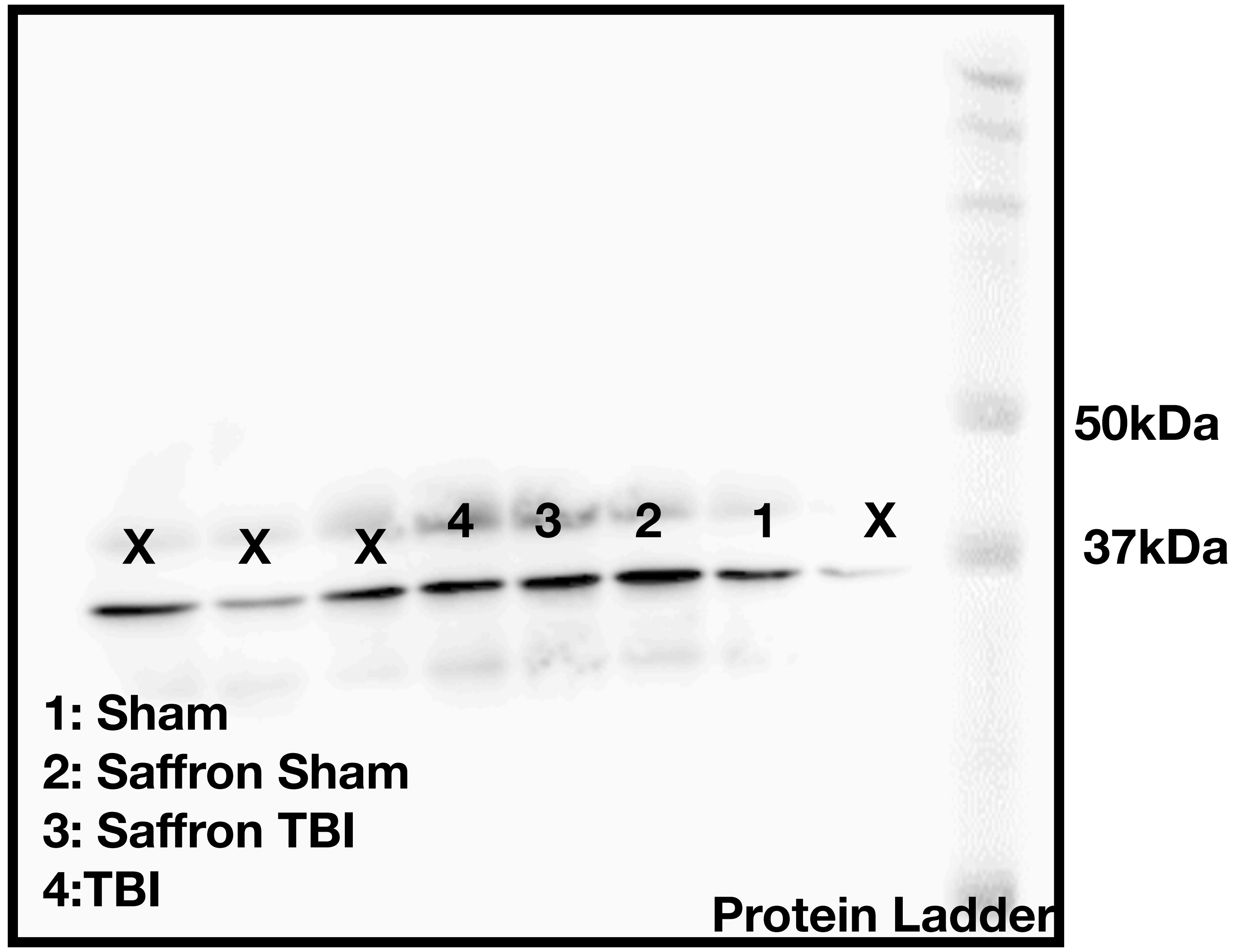

NLRP3 Original Blot Image

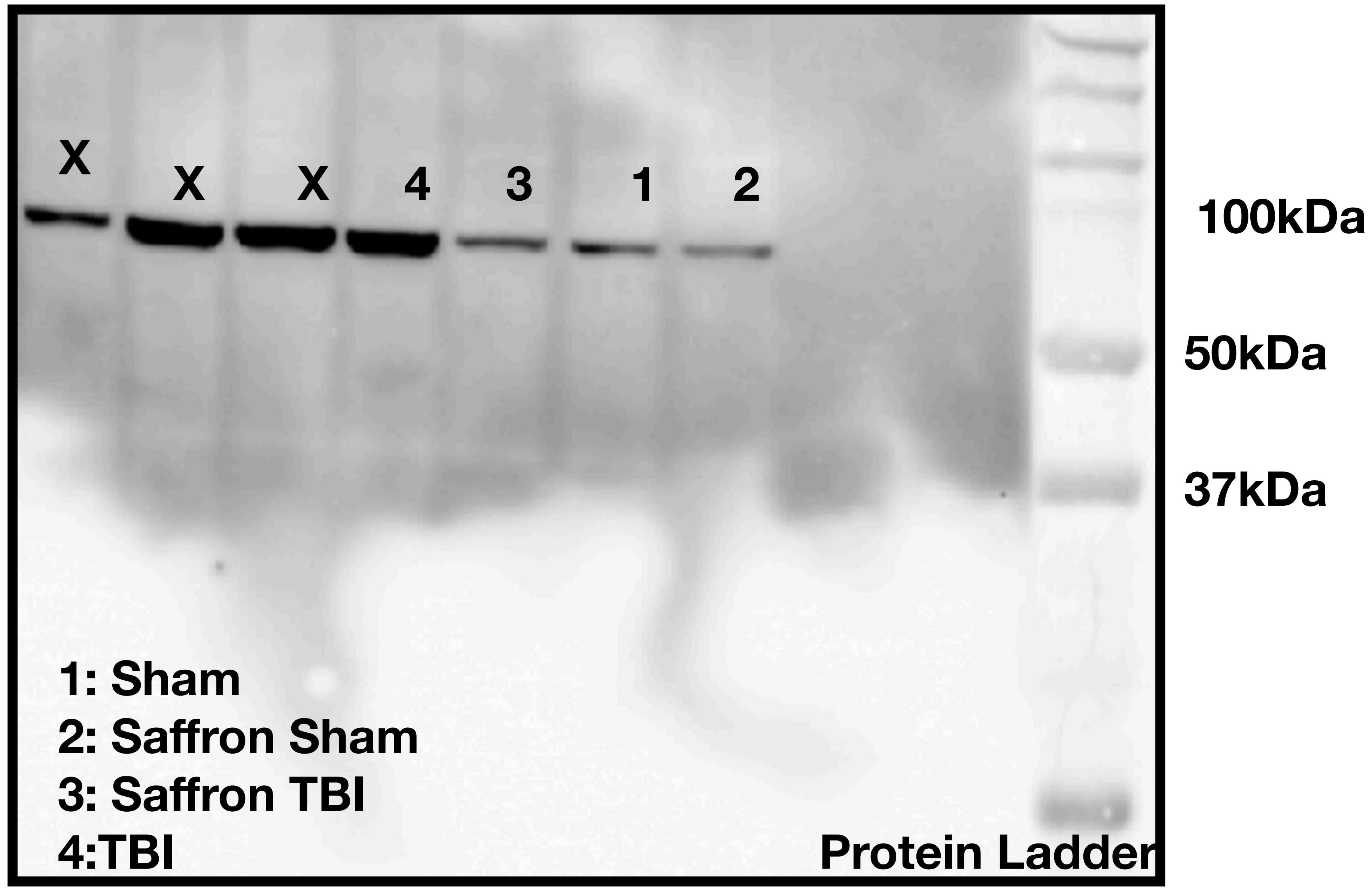

SIRT1 Original Blot Image

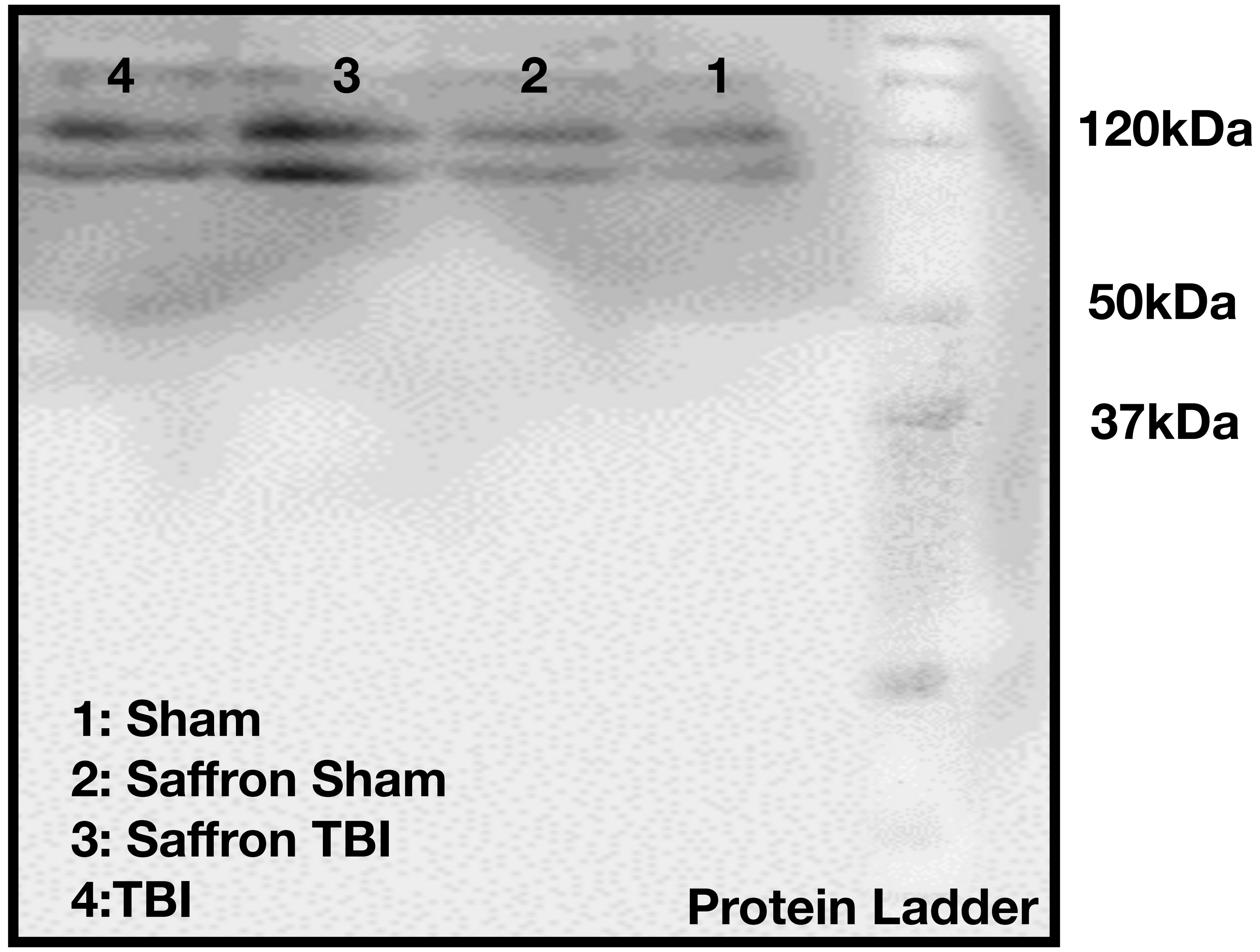

GFAP Original Blot Image

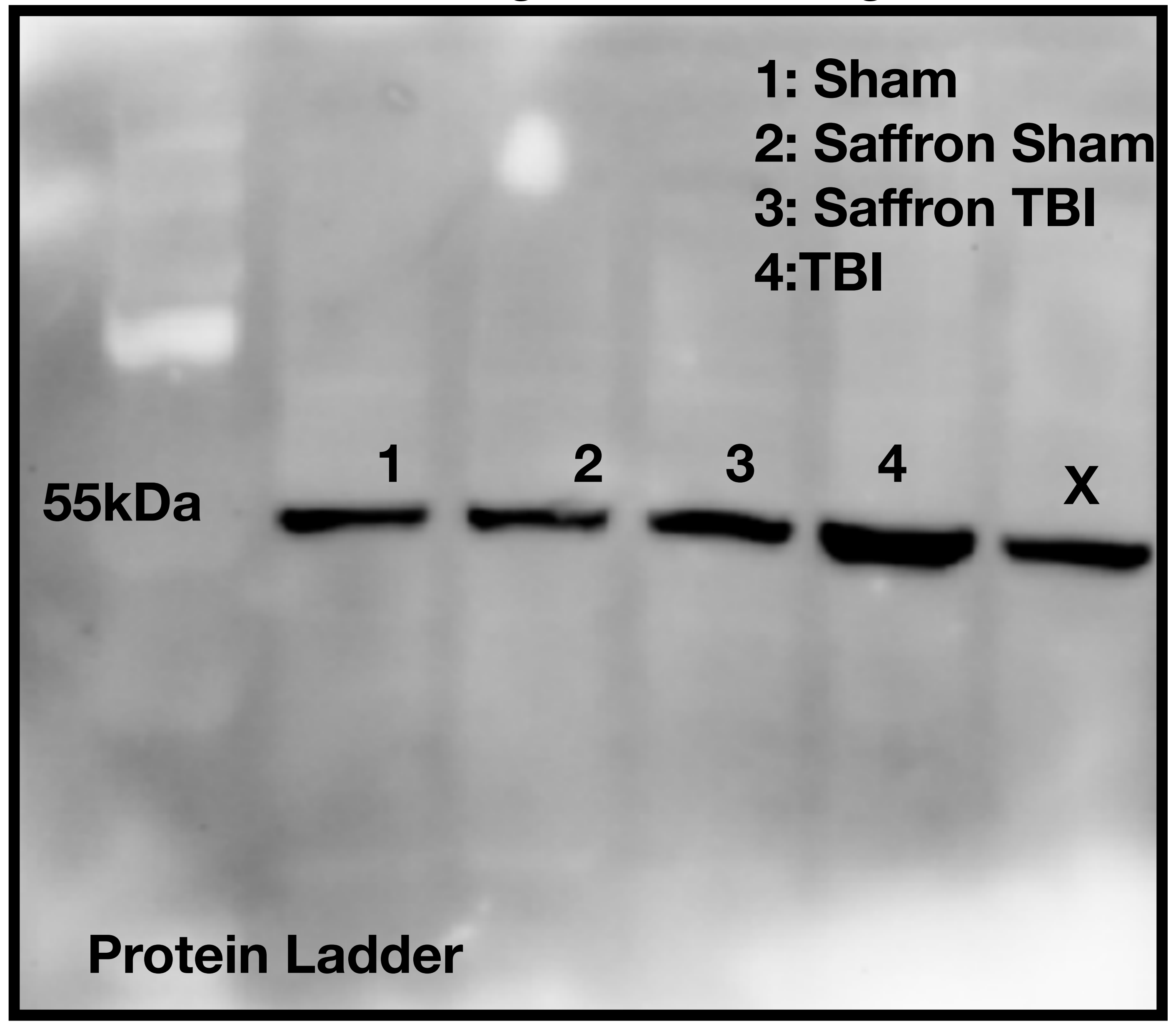

Beta tubulin Original Blot Image

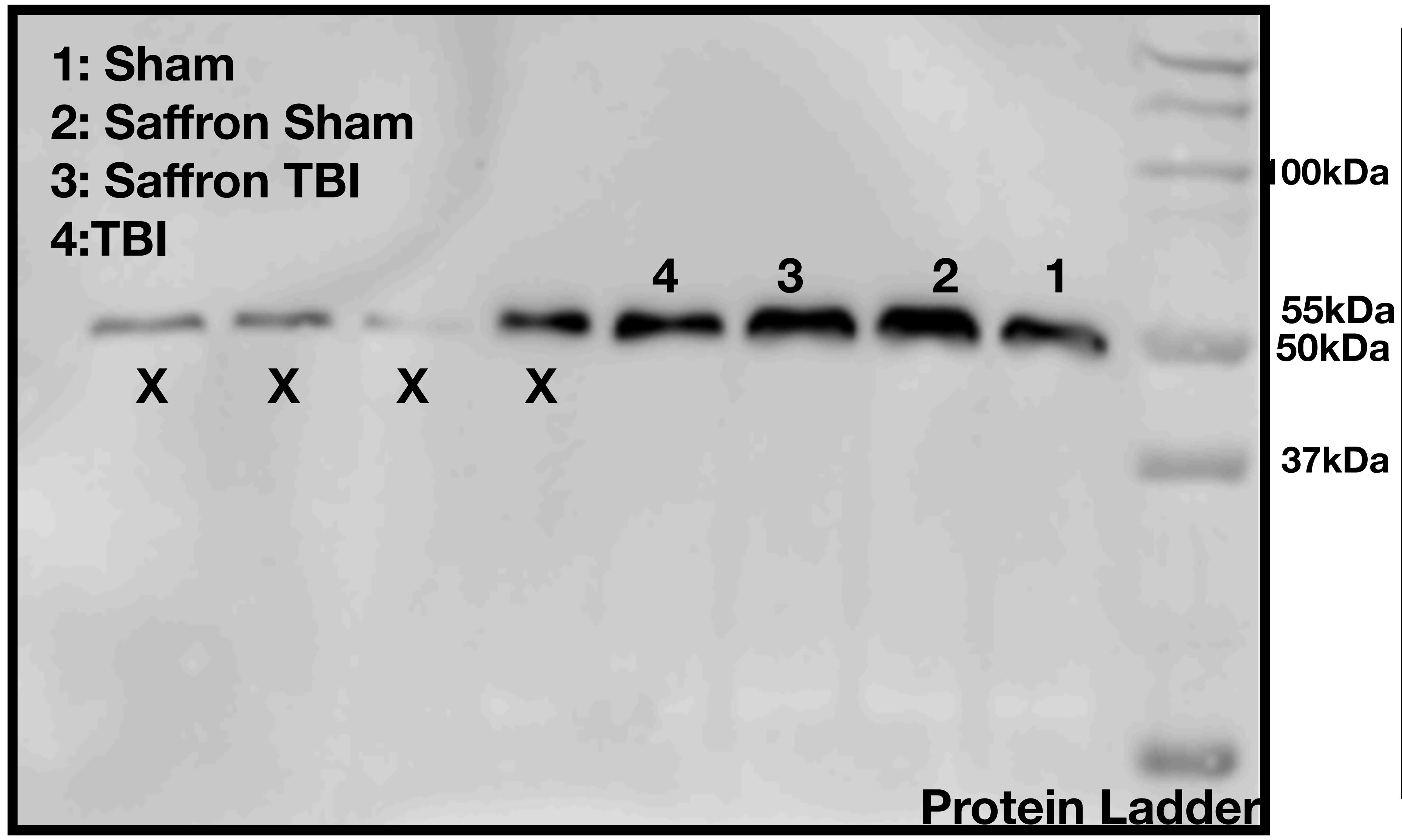

IBA1 Original Blot Image

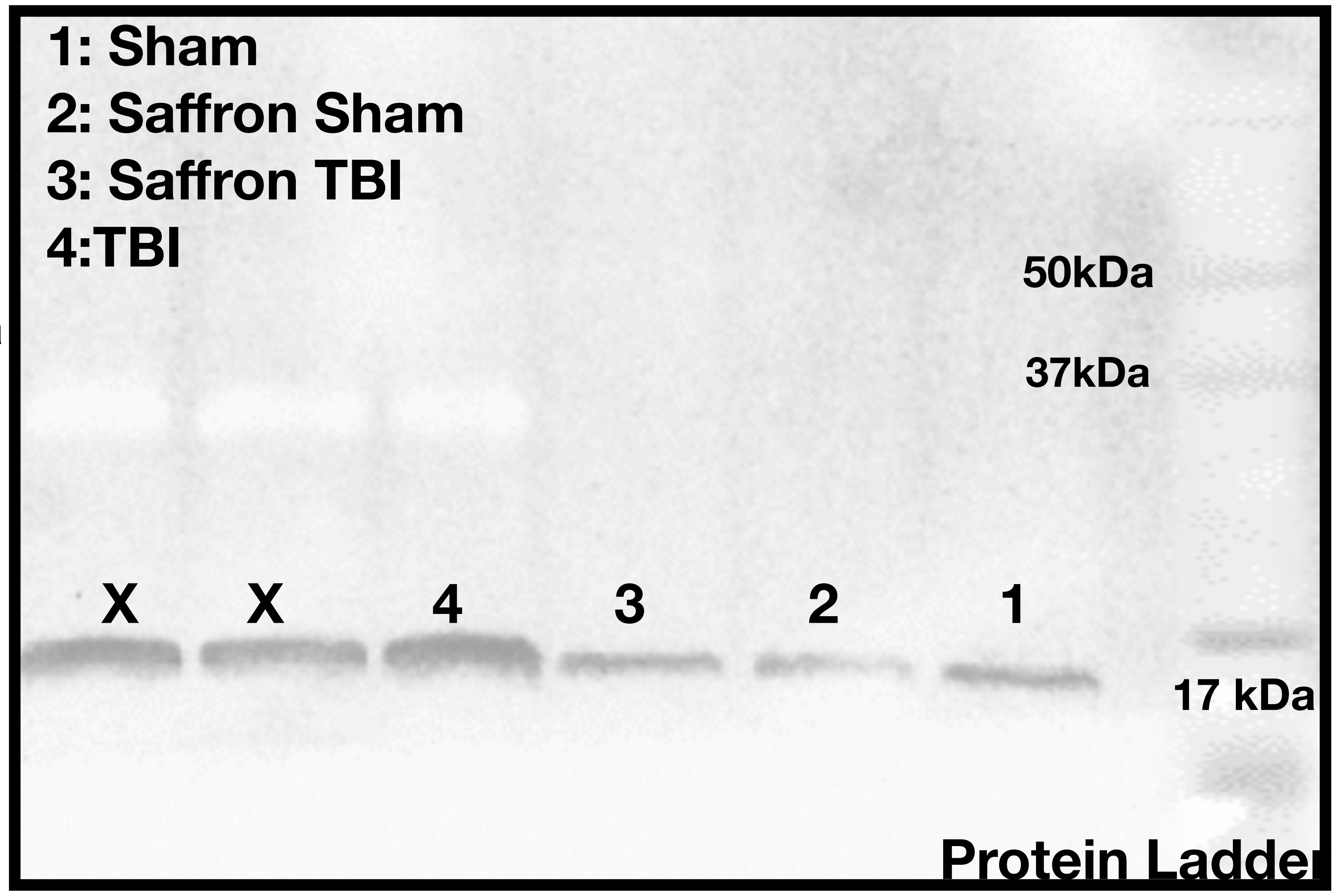

NeuN Original gel

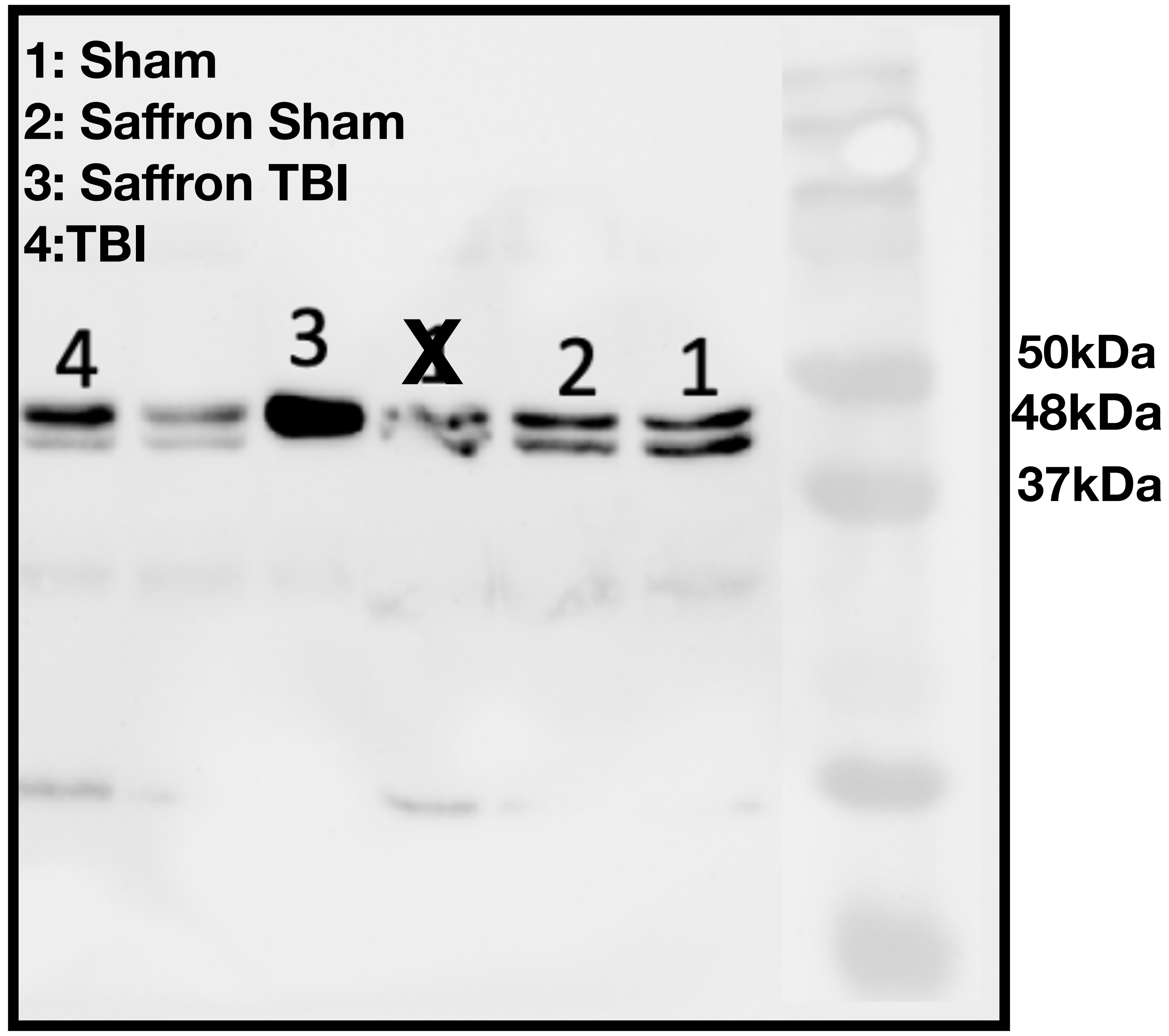

Any lane with X symbol is not represented in the figure submitted within the manuscript, we aimed to choose 4 consecutive bands that represent the 4 different experimental groups
