# Supplementary material for: Saffron extract attenuates neuroinflammation in rmTBI mouse model by suppressing NLRP3 inflammasome activation via SIRT1
Source: PLoS One. 2021 Sep 10;16(9):e0257211. doi: 10.1371/journal.pone.0257211 (PMC8432768; doi:10.1371/journal.pone.0257211)
Supplement: Attachment — Submitted filename: RESPONSE TO REVIEWERS .docx [file pone.0257211.s002.docx]

**RESPONSE TO REVIEWERS**

**of “Saffron Extract Attenuates Neuroinflammation in rmTBI Mouse Model by Suppressing NLRP3 Inflammasome Activation via SIRT1”**

**Manuscript ID: PONE-D-21-13059**

**by Mariam Shaheen, Amira Bekdash, Hana A. Itani, Jamilah Borjac**

We would like to thank the reviewers for their thorough review of the manuscript as they raise important issues and their comments are very helpful for improving the manuscript. We agree with almost all their comments and we have revised our manuscript accordingly. We included all reviewers’ suggestions and clarified the text when needed. We are confident that the new version of the manuscript is greatly improved. We respond below in detail to each of the reviewer’s comments.

In addition, we hope that the reviewers find our responses to their comments satisfactory. Please, find below the reviewers comments repeated in italics and our responses inserted after each comment in italic blue.

Looking forward to hearing from you soon.

Sincerely,

Authors

**Response to comments**

*1.	Among the overarching concerns, the statistical analysis lacks sufficient detail, testing whether interaction effects between groups for the various outcome measures are significant. The results for* *the interaction terms in the overall ANOVAs are important to confirm that the effects on the various outcome measures of interest are in fact saffron-dependent, as the report implies.*

***Reply:*** *The authors did the needed corrections and added the related statistical data (Interaction F test) in the Result section in the following paragraphs/lines:*

## ***Effect of Saffron on Bodyweight/ Lines 252-254***

## ***Saffron extract reduces protein expression levels of GFAP, NeuN and Iba1 in the injured cortex following rmTBI /Lines 283-284***

- ***Saffron pretreatment reduced the mRNA expression and protein levels of NLRP3 in the injured cortex following rmTBI/ Lines 308***
- ***Saffron pretreatment reduced the mRNA expression of NLRP3 associated genes ASC, caspase 1, IL‐1β, and IL‐18 in the injured cortex following rmTBI /Lines 326-328, 335***
- ***Saffron extract attenuated NLRP3 inflammasome signaling activation via enhanced expression of SIRT1 in the injured cortex following rmTBI/ Lines 353, 355***
- ***Saffron extract upregulates mRNA expression of NRF2 and heme oxygenase-1 (HMOX1) in the injured cortex following rmTBI/ Lines 377,379***

*2.	An open problem is to understand how saffron metabolites reach the CNS after i.p. application, is this a passive transcellular diffusion or an active one?*

***Reply:*** *Saffron’s two major carotenoids, crocin and crocetin are the most biologically active elements. The primary site for carotenoids metabolism is the intestinal tract (1). In the intestine, crocin in the saffron is deglycosylated to trans-crocetin (2) enzymatically by the fecal microbiota (3). Trans-crocetin is then absorbed into the bloodstream via passive transcellular diffusion (3). Crocetin, in the bloodstream, is bound to albumin. The albumin-bound crocetin pushes through the blood brain barrier slowly enough to enter the CNS (3).*

***The authors added the above information in the Introduction section lines (103-108)***

***References:***

*1. Parker RS. 1996. “Absorption, Metabolism, and Transport of Carotenoids.” FASEB J. 10(5):542–51.*

*2. Lautenschläger, M., J. Sendker, S. Hüwel, H. J. Galla, S. Brandt, M. Düfer, K. Riehemann, and A. Hensel. 2015. “Intestinal Formation of Trans-Crocetin from Saffron Extract (Crocus Sativus L.) and in Vitro Permeation through Intestinal and Blood Brain Barrier.” Phytomedicine 22(1). doi: 10.1016/j.phymed.2014.10.009.*

*3. Asai, Akira, Takahisa Nakano, Masahiro Takahashi, and Akihiko Nagao. 2005. “Orally Administered Crocetin and Crocins Are Absorbed into Blood Plasma as Crocetin and Its Glucuronide Conjugates in Mice.” Journal of Agricultural and Food Chemistry 53(18). doi: 10.1021/jf0509355*

*3.	It is not clear from the materials and methods which dose/volume of Saffron-Extract was i.p injected. It is mentioned on page 4 …the dose of the saffron extract used in this study was based on previous studies 32, 33???*

***Reply:*** *The dose of saffron extract used was 50mg/kg. Animal studies on saffron, showed that a dose of 50mg/kg of aqueous saffron extract was safe and effective (4), and considered within the range of safe doses used in neurological related disorders and other diseases (4-9).*

***The authors added the dose used with all the related in references in the manuscript, Material and Method section, paragraph 1 under “Preparation of saffron water extract” lines (140-142).***

***Reference:***

4.	José Bagur, M. *et al.* Saffron: An Old Medicinal Plant and a Potential Novel Functional Food. *Molecules* **23**, (2017).

5.	Amin, B., Moghri Feriz, H., Timcheh Hariri, A., Tayyebi Meybodi, N. & Hosseinzadeh, H. Protective effects of the aqueous extract of Crocus sativus against ethylene glycol induced nephrolithiasis in rats. *EXCLI Journal* **14**, 411–422 (2015).

6.	Hosseinzadeh, H., Abootorabi, A. & Sadeghnia, H. R. Protective Effect of Crocus sativus Stigma Extract and Crocin ( trans-crocin 4) on Methyl Methanesulfonate–Induced DNA Damage in Mice Organs. *DNA and Cell Biology* **27**, 657–721 (2008).

7. Ettehadi, H. *et al.* Aqueous Extract of Saffron (Crocus sativus) Increases Brain Dopamine and Glutamate Concentrations in Rats. *Journal of Behavioral and Brain Science* **03**, (2013).
8. Bostan, H. B., Mehri, S. & Hosseinzadeh, H. Toxicology effects of saffron and its constituents: A review. *Iranian Journal of Basic Medical Sciences* **20**, 110–121 (2017).
9. Ziaee, T., Razavi, B. M. & Hosseinzadeh, H. Saffron Reduced Toxic Effects of its Constituent, Safranal, in Acute and Subacute Toxicities in Rats. *Jundishapur Journal of Natural Pharmaceutical Products* **9**, 3–8 (2013).
4. *Neuronal severity score: why the data are not shown?*

***Reply:*** *The results of the neuronal severity score (NSS) along with other behavioral tests are part of another manuscript submitted to Scientific Reports and is currently under revision. Calculation of the NSS was based on Zhuang et al. (10). The NSS was assessed at 1hour and 24hours post the final injury. The difference between the NSS at two different time points is a parameter that reflects injury severity post-TBI, as described by Chen et al. (11). The scores were classified as follows: 13-18 severe injury, 7-12 moderate injury,1-6 mild injury.*

***References:***

10. *Zhuang, Jian, Jie Li, Evelyn Ooi, Jonathan Bloom, Carrie Poon, Lax Daniel, Rosenbaum Daniel M, and Barone Frank C. 2015. “Modified Neurological Severity Score (MNSS) Tests and Scoring Values.” PLOS ONE.Dataset.*
11. *Chen, Yun, Shlomo Constsntini, Victoria Trembovler, Marta Weinstock, and Esther Shoham. 1996. “An Experimental Model of Closed Head Injury in Mice: Pathophysiology, Histopathology, and Cognitive Deficits.” Journal of Neurotrauma 13(10). doi: 10.1089/neu.1996.13.557.*
5. *In Hits 1 and 2, the differences between Sham/saffron sham and TBI are not significant, however they are significant in Hits 5, 6 and 7. How to explain that TBI group failed to maintain normal body weights and Saffron-TBI mice were able to gain weight. Is this due to the injury or to saffron. One can be assumed that injured mice have motoric difficulties to food-access.*

***Reply:*** *In a separate study under this project, we tested the motor behaviors of TBI and Saffron TBI groups using the NSS score and other motor tests such as rotarod test, and pole climb test. Our results showed that the TBI injury caused motor deficits and that saffron-treatment restored motor functions. Despite the fact that saffron has proved to be satiety enhancer (12), reduces blood biomarkers associated with obesity (13), and exert anti-obesity and body weight management effects in rats fed with high fat diet (14), treating TBI mice with saffron allowed weight-gain due to its neuroprotective effect that aided in restoring motor deficits and thus facilitating their access to food. The authors emphasized on this comment in the Discussion section, lines 412-418.*

***Reference:***

*12.	Gout, B., Bourges, C. & Paineau-Dubreuil, S. Satiereal, a Crocus sativus L extract, reduces snacking and increases satiety in a randomized placebo-controlled study of mildly overweight, healthy women. Nutrition Research* ***30****, (2010).*

*13.	Ramli, F. N., Abu Bakar Sajak, A., Abas, F., Mat Daud, Z. A. & Azlan, A. Effect of Saffron Extract and Crocin in Serum Metabolites of Induced Obesity Rats. BioMed Research International* ***2020****, (2020).*

*14.	Mashmoul, M. et al. Effects of saffron extract and crocin on anthropometrical, nutritional and lipid profile parameters of rats fed a high fat diet. Journal of Functional Foods* ***8****, (2014).*

6. *In Hit1 TBI- 35 vs TBI+Saffron 32 this difference is not significant- Hit5: TBI approx. 32- TBI+Saffron 31 this difference is significant (p< 0.0001). Is it quite difficult to understand this statistic interpretation? The same remark for Hits 6 & 7.*

***Reply:*** *The determination of the p value depends on the average and the standard deviation* *of the variable tested. Therefore, based on the weigh averages and their SD of all groups, data analysis showed significant difference only in hit 5, 6 and 7.*

7. *The weight differences are in the gram range; it cannot be ruled out that the edema could play a role. While Brain edema is a severe complication of TBI. The accumulation of intracellular water, especially in the perivascular astrocytes, disrupts the local osmotic environment, resulting in or aggravating the breakdown of the BBB.*

***Reply:*** *Yes of course, brain edema is among the well noted features post brain injury. Unfortunately, we didn’t measure the total brain weight directly after dissection, but from our histological examination (results in the submitted manuscript to Scientific Reports under revision) edema was not noted which means the weight difference observed is not due to edema.*

8. *Authors stated that saffron extract reduces neuronal damage, astrocyte’s proliferation and microglial activation which are TBI-mediated. This analysis has been done only on proteins-levels by WB. It should be emphasized that the increase in GFAP- NeuN- and Iba1-Levels does not mean automatically glia-proliferation, neuronal-protection and microglia-activation! These changes may indicate only alterations in protein-levels and not inevitably changes in the density of astrocytes, neurons and microglial-cells. Morphological data are needed in order to show that saffron reduced the TBI-mediated astrogliosis, neuronal damage and microglia activation.*

## ***Reply:*** *We totally agree with the reviewers that WB data reflects alteration in proteins-levels and more experiments are needed. However, one of the major limitations to any study is the budget allocated. In this study, the first author had to pay for the majority of the kits needed in the project where this study constitutes only one part. Currently, the Lebanese currency crisis made it extremely difficult to add any additional experiments to relate the western blot results to immunohistochemistry studies or morphological studies. Based on the reviewer comment, we changed, in the Result section, the tittle of the paragraph entitled with “****Saffron extract reduced neuronal damage, astrocyte and microglial activation in the injured cortex following rmTBI****” to* ***“Saffron extract reduces GFAP, NeuN and Iba1 protein levels in the injured cortex following rmTBI****”, and emphasized within the same paragraph, lines 279-281, and 286-287, that western blot results on GFAP, NeuN, and Iba1-Levels does not necessarily reflect glia-proliferation, neuronal-protection and microglia-activation.*

9. *Furthermore, recent studies investigating neurodegenerative diseases reveals that neuroinflammation in the CNS induces the activation of two different paradigm of reactive astrocytes, termed A1 and A2. Notably, astrocyte reactivity is disease- and stimulus-dependent, adopting either a cytotoxic A1 phenotype or a neurotrophic, anti-inflammatory A2 phenotype. This point might be discussed.*

***Reply: The answer to the above comments is added in the Discussion section lines (420-440) as follow:***

*“The glial cells, microglial cells and astrocytes are the resident cells in the CNS. They arise in response to TBI to exert their neuroprotective effects to improve TBI outcomes through their interactions. Reactive astrocytes can be classified into A1 and A2 phenotypes that provide neuroprotective and neurotoxic effects respectively. As part of its neuroprotective role, astrocytes, first can lessen glutamate excitotoxicity by reducing extracellular levels of glutamate (12), and enhance expression of neurotrophic factors such as brain-derived neurotrophic factor (BDNF) to reduce the injury-induced neuronal death, increase cell proliferation, and axonal repair (13). Astrocytes can also regulate the ionic balance and cerebral blood flow (14), provide substrates needed in energy production for neurons (15), play a role in synapse development, and repair neuronal work (16). Once TBI occurs, astrocytes are among the primary cells that respond through astrogliosis (17). The reaction is linked to cellular proliferation and hypertrophy along with increment levels in vimentin and GFAP (18). It also causes excessive production of matrix metalloprotease (MMP) that can affect BBB structure (19), and aquaporin 4 protein that induces edema (20). Glial scar formation is also contributed to astrocyte, where after TBI, hypertrophic astrocytes are recruited to the site of damage, secrete the proteoglycan chondroitin sulfate, an inhibitory cellular matrix (21). The secreted matrix forms a physical and chemical barrier that can protect healthy brain tissue from the neurotoxins of the injury section, but this barrier still inhibits axonal repairment and growth (22). Although astrogliosis is vital for axonal growth following the injury, the timeline of injury progression controls the advantages and disadvantages of astrogliosis to the CNS cells (23), where prolonged astrocytes reactivity may hinder axonal regeneration and functional recovery (22).*

***Reference:***

*12.	Sofroniew M v. Astrocyte barriers to neurotoxic inflammation. Nature Reviews Neuroscience. 2015 May 20;16(5).*

*13.	Zou P, Liu X, Li G, Wang Y. Resveratrol pretreatment attenuates traumatic brain injury in rats by suppressing NLRP3 inflammasome activation via SIRT1. Molecular Medicine Reports. 2018;17(2):3212–7.*

*14.	Baldwin KT, Eroglu C. Molecular mechanisms of astrocyte-induced synaptogenesis. Current Opinion in Neurobiology. 2017 Aug;45.*

*15.	Burda JE, Bernstein AM, Sofroniew M v. Astrocyte roles in traumatic brain injury. Experimental Neurology. 2016 Jan;275.*

*16.	Luo H, Wu X-Q, Zhao M, Wang Q, Jiang G-P, Cai W-J, et al. Expression of vimentin and glial fibrillary acidic protein in central nervous system development of rats. Asian Pacific Journal of Tropical Medicine. 2017 Dec;10(12).*

*17.	Cabezas R, Ãvila M, Gonzalez J, El-BachÃ¡ RS, BÃ¡ez E, GarcÃ­a-Segura LM, et al. Astrocytic modulation of blood brain barrier: perspectives on Parkinsons disease. Frontiers in Cellular Neuroscience. 2014 Aug 4;8.*

18.	*Saadoun S. Involvement of aquaporin-4 in astroglial cell migration and glial scar formation. Journal of Cell Science. 2005 Dec 15;118(24).*

*19.	Cafferty WBJ, Yang S, Duffy PJ, Li S, Strittmatter SM. Functional Axonal Regeneration through Astrocytic Scar Genetically Modified to Digest Chondroitin Sulfate Proteoglycans. The Journal of Neuroscience. 2007;27(9):2176–85.*

*20.	Sofroniew M v. Molecular dissection of reactive astrogliosis and glial scar formation. Trends in Neurosciences. 2009;32(12):638–47.*

*21.	Toy D, Namgung U. Role of Glial Cells in Axonal Regeneration. Experimental Neurobiology. 2013 Jun 30;22(2).*

*22.	Morganti-Kossmann MC, Semple BD, Hellewell SC, Bye N, Ziebell JM. The complexity of neuroinflammation consequent to traumatic brain injury: from research evidence to potential treatments. Acta Neuropathologica. 2019 May 7;137(5).*

10. *How to explain the significant increase in NeuN-levels in the TBI-group comparing to sham-group on Fig. 3? If TBI induced neuronal injury, we should expect a decrease in neuronal density and consequently a reduction of NeuN-protein-levels!*

***Reply:*** *In our work we assessed the NeuN protein level 24h post the final injury and as far as we know, no studies assessed the protein level at this time point. The increased NeuN levels was observed in both TBI and Saffron TBI group. This finding needs further analysis and experimentation. In our model which is repetitive injury with only 48h interval between each hit, the injured neurons may attempt to compensate through its membrane capability of resealing all over the period of injury. We completely agree that western blot per se is not enough to confirm neuronal injury, however we thought that this distinctive notable increase of NeuN in TBI group and Saffron TBI must be mentioned and that it may open the way for new insights on understanding NeuN roles.*

11. *SIRT1- was significantly increased in TBI comparing to sham, but instead of normalization, we see a potentiation of SIRT-1 levels in saffron-TBI-Group. This is an interesting finding which needs a confirmation by using pharmacological inhibitors of saffron-uptake.*

***Reply:*** *The main aim of our current study was to assess NLRP3 levels post rmTBI and examine the potential effects of saffron extract and the possible role or involvement of SIRT1 in this pathway. We totally agree with the reviewer that it would be quite interesting to find that saffron enhances SIRT1 expression and instead of normalization, it potentiated SIRT-1 levels in saffron-TBI-Group. Sirtinol which is a pharmacological inhibitor of saffron uptake could be used in later experiments to prove this point as used by Yang et al who showed the effect of SIRT1 in TBI (23). However, in our study, the Saffron sham group received saffron in the absence or occurrence of injury and both the saffron sham and sham group showed similar immunoblot results. Thus, we can assume that saffron by itself does not induce the increase in SIRT1 expression unless tissue damage occurs, i.e. post the injury. Using the pharmacological inhibitor would clarify the interplay between saffron, TBI, and SIRT1.*

**Reference:**

23. Yang, H. *et al.* SIRT1 plays a neuroprotective role in traumatic brain injury in rats via inhibiting the p38 MAPK pathway. *Acta Pharmacologica Sinica* **38**, 168–181 (2017).

12. *The same evolution concerns NRF2 and Hmox1 on mRNA-levels on Fig. 7. Here, why the data show only mRNA- and not protein-levels.*

***Reply:*** *Quantifying the protein levels of both NRF2 and Hmox would definitely add more values to our results. However, due to the Lebanese currency crisis and the limited funds available, protein quantification was not performed.*

***Reviewer #2:***

*The manuscript, was found to be interesting on the exploring the anti-neuro-inflammatory effect of aquas (polar) extract if the authors would have explained on mode of action on the base of compound level in the saffron extract, it will be more interesting and information on compound level. For example, at least partial purification based such as either it is a protein or peptide or either it is a polyphenol-based compound it will be much more informative on the effect of the extract. In efficacy and durability of the anti-neuroinflammatory property depends on the nature of the compound present in the saffron extract.*

***Reply: The authors have added in the introduction section, line “99-102” and lines” 110-112” the following paragraph describing saffron major components, and the anti-inflammatory properties Saffron and its main active constituents****.*

*“Phytochemical studies on saffron showed four major metabolites, crocin (80%), safranal (70%), and a modest percentage of picrocrocin and crocetin. The chemical analysis also revealed over 200 distinct compounds found in saffron including flavonoids, amino acids, vitamins (riboflavin and thiamine), anthocyanin, proteins, α-carotene, β-carotene, starch, zeaxanthin, mineral matter, and gums (24).*

# *“Saffron exhibits antioxidant properties (25) and anti-inflammatory activities (26) via its major metabolites crocin (27,28), crocetin (29), and safranal (30).*

*However, we believe that attempting to determine the effect of isolated compounds would be the scope of a new study.*

***References:***

24.	Alavizadeh, S. H. & Hosseinzadeh, H. Bioactivity assessment and toxicity of crocin: A comprehensive review. *Food and Chemical Toxicology* **64**, (2014).

25.	Yoshino, F., Yoshida, A., Umigai, N., Kubo, K. & Chang, M. Crocetin reduces the oxidative stress induced reactive oxygen species in the stroke prone spontaneously hypertensive rats ( SHRSPs ) brain. *J Clin Biochem Nutr* **49**, 182–187 (2011).

26.	Christodoulou, E., Kadoglou, N. P., Kostomitsopoulos, N. & Valsami, G. Saffron: A natural product with potential pharmaceutical applications. *Journal of Pharmacy and Pharmacology* **67**, 1634–1649 (2015).

27.	Kim, J.-H. *et al.* Crocin Suppresses LPS-Stimulated Expression of Inducible Nitric Oxide Synthase by Upregulation of Heme Oxygenase-1 via Calcium/Calmodulin-Dependent Protein Kinase 4. *Mediators of Inflammation* **2014**, (2014).

28.	Xiong, Y., Wang, J., Yu, H., Zhang, X. & Miao, C. Anti-asthma potential of crocin and its effect on MAPK signaling pathway in a murine model of allergic airway disease. *Immunopharmacology and Immunotoxicology* **37**, (2015).

29.	Diao, M., Min, J., Guo, F. & Zhang, C.-L. Effects of salbutamol aerosol combined with magnesium sulfate on T-lymphocyte subgroup and Th1/Th2 cytokines of pediatric asthma. *Experimental and Therapeutic Medicine* **13**, (2017).

30.	Farokhnia, M. *et al.* Comparing the efficacy and safety of Crocus sativus L. with memantine in patients with moderate to severe Alzheimer’s disease: a double-blind randomized clinical trial. *Human Psychopharmacology: Clinical and Experimental* **29**, (2014).
