# Supplementary material for: Saffron extract attenuates neuroinflammation in rmTBI mouse model by suppressing NLRP3 inflammasome activation via SIRT1
Source: PLoS One. 2021 Sep 10;16(9):e0257211. doi: 10.1371/journal.pone.0257211 (PMC8432768; doi:10.1371/journal.pone.0257211)
Supplement: Attachment — Submitted filename: RESPONSE TO REVIEWERS.docx [file pone.0257211.s003.docx]

**RESPONSE TO REVIEWERS**

**of “Saffron Extract Attenuates Neuroinflammation in rmTBI Mouse Model by Suppressing NLRP3 Inflammasome Activation via SIRT1”**

**Manuscript ID: PONE-D-21-13059**

**by M. Shaheen, A. Bekdash, H. Itani, and J. Borjac**

We would like to thank the reviewers for their thorough review of the manuscript as they raise important issues and their comments are very helpful for improving the manuscript. We agree with almost all their comments, and we have revised our manuscript accordingly. We crafted a revised version of the paper that states the hypothesis and the implications of our work more clearly than before. Moreover, we included all reviewers’ suggestions and clarified the text when needed. We are confident that the new version of the manuscript will be greatly improved. We respond below in detail to each of the reviewer’s comments.

In addition, we hope that the reviewers find our responses to their comments satisfactory. Please, find below the reviewers' comments repeated in italics and our responses inserted after each comment in italic blue.

Looking forward to hearing from you soon.

Sincerely,

Authors

*Reviewer #1: Given financial problems as a reason for not having done this or that experiment is not logical in the scientific world. It is always possible to do so in a collaborative framework. In any case, one should never hesitate to ask for help elsewhere from colleagues who have the expertise and the means. I am willing to do it for you, but it seems that the authors are eager to see their paper published. In any case, the authors have taken my criticism into consideration, which collectively improve the quality of the manuscript.*

*Reply: We would like to thank reviewer # 1 and we totally agree about the collaborative work between researchers. Indeed this work is a collaboration between Beirut Arab University and the American University of Beirut. We are indeed eager to publish this work as it is one of the graduation requirements for the first author of this manuscript who is a Ph.D. student.*

*Reviewer #3:*

*The manuscript needs considerable proofreading and editing, which should not be the responsibility of a reviewer.*

*Reply: The authors made all the needed editing*

*A detailed description of the saffron extract injected. What exactly did the authors inject? The aqueous 2% solution, the freeze-dried precipitate, or the re-solubilized freeze-dried precipitate? Please try to be more clear in the description. Most ingredients in saffron won’t be water-soluble, please discuss this to allow speculation which chemical substance in saffron might be responsible for neuroprotection.*

*Reply: A 2% aqueous solution was prepared by soaking one gram of ground saffron stigmas in 50ml of distilled water for 3 hours at room temperature in the dark. The filtrate was then freeze-dried yielding 0.065 g of a yellow-orange precipitate The precipitate was suspended in saline to a final concentration of 8.6% and used at a dose of 50mg/kg of body weight. The saffron dose used was based on animal studies showing its safety and effectiveness in neurologically related disorders and other diseases* [1–6]*. The phytochemical studies on saffron showed four major metabolites, crocin (80%), safranal (70%), and a modest percentage of picrocrocin and crocetin. These primary components modulate and manipulate saffron pharmacological effects and nutritional properties* [7]*. Yes, as the reviewer stated most ingredients in saffron are not water-soluble, but crocin is water-soluble. Crocin (C_44_H_70_O_28_), is the saffron’s major water-soluble stable carotenoid* [8]*. Many studies investigated and proved the neuroprotective effect of crocin in the TBI model* [9]*, Alzheimer’s disease* [10]*, neurotoxicity* [11]*, cerebral ischemia* [12]*, and in many different neurological disorders* [13–18]*.*

*Reference:*

*1.	Amin B, Moghri Feriz H, Timcheh Hariri A, Tayyebi Meybodi N, Hosseinzadeh H. Protective effects of the aqueous extract of Crocus sativus against ethylene glycol induced nephrolithiasis in rats. EXCLI Journal. 2015;14: 411–422. doi:10.17179/excli2014-510*

*2.	Hosseinzadeh H, Abootorabi A, Sadeghnia HR. Protective Effect of Crocus sativus Stigma Extract and Crocin ( trans-crocin 4) on Methyl Methanesulfonate–Induced DNA Damage in Mice Organs. DNA and Cell Biology. 2008;27: 657–721. doi:10.1089/dna.2008.0767*

*3.	Ettehadi H, Mojabi SN, Ranjbaran M, Shams J, Sahraei H, Hedayati M, et al. Aqueous Extract of Saffron (Crocus sativus) Increases Brain Dopamine and Glutamate Concentrations in Rats. Journal of Behavioral and Brain Science. 2013;03. doi:10.4236/jbbs.2013.33031*

*4.	Bostan HB, Mehri S, Hosseinzadeh H. Toxicology effects of saffron and its constituents: A review. Iranian Journal of Basic Medical Sciences. 2017;20: 110–121. doi:10.22038/ijbms.2017.8230*

*5.	Ziaee T, Razavi BM, Hosseinzadeh H. Saffron Reduced Toxic Effects of its Constituent, Safranal, in Acute and Subacute Toxicities in Rats. Jundishapur Journal of Natural Pharmaceutical Products. 2013;9: 3–8. doi:10.17795/jjnpp-13168*

*6.	José Bagur M, Alonso Salinas G, Jiménez-Monreal A, Chaouqi S, Llorens S, Martínez-Tomé M, et al. Saffron: An Old Medicinal Plant and a Potential Novel Functional Food. Molecules. 2017;23. doi:10.3390/molecules23010030*

*7.	Pandita D. Saffron (Crocus sativus L.): phytochemistry, therapeutic significance and omics-based biology. Medicinal and Aromatic Plants. Elsevier; 2021. doi:10.1016/B978-0-12-819590-1.00014-8*

*8.	Alavizadeh SH, Hosseinzadeh H. Bioactivity assessment and toxicity of crocin: A comprehensive review. Food and Chemical Toxicology. 2014;64. doi:10.1016/j.fct.2013.11.016*

*9.	Wang K, Zhang L, Rao W, Su N, Hui H, Wang L, et al. Neuroprotective effects of crocin against traumatic brain injury in mice: Involvement of notch signaling pathway. Neuroscience Letters. 2015;591: 53–58. doi:10.1016/j.neulet.2015.02.016*

*10.	Lin L, Liu G, Yang L. Crocin Improves Cognitive Behavior in Rats with Alzheimer’s Disease by Regulating Endoplasmic Reticulum Stress and Apoptosis. BioMed Research International. 2019;2019: 1–9. doi:10.1155/2019/9454913*

*11.	Rao SV, Hemalatha P, Yetish S, Muralidhara M, Rajini PS. Prophylactic neuroprotective propensity of Crocin, a carotenoid against rotenone induced neurotoxicity in mice: behavioural and biochemical evidence. Metabolic Brain Disease. 2019;34. doi:10.1007/s11011-019-00451-y*

*12.	Zheng Y-Q, Liu J-X, Wang J-N, Xu L. Effects of crocin on reperfusion-induced oxidative/nitrative injury to cerebral microvessels after global cerebral ischemia. Brain Research. 2007;1138. doi:10.1016/j.brainres.2006.12.064*

*13.	Shaterzadeh-Yazdi H, Samarghandian S, Farkhondeh T. Effects of Crocins in the Management of Neurodegenerative Pathologies: A Review. Neurophysiology. 2018;50: 302–308. doi:10.1007/s11062-018-9752-0*

*14.	Farkhondeh T, Samarghandian S, Yazdi HS, Samini F. The protective effects of crocin in the management of neurodegenerative diseases: a review. Am J Neurodegener Dis. 2018. Available: www.AJND.us*

*15.	Zhang X, Zhang X, Dang Z, Su S, Li Z, Lu D. Cognitive Protective Mechanism of Crocin Pretreatment in Rat Submitted to Acute High-Altitude Hypoxia Exposure. BioMed Research International. 2020;2020. doi:10.1155/2020/3409679*

*16.	Yuan Y, Shan X, Men W, Zhai H, Qiao X, Geng L, et al. The effect of crocin on memory, hippocampal acetylcholine level, and apoptosis in a rat model of cerebral ischemia. Biomedicine & Pharmacotherapy. 2020;130. doi:10.1016/j.biopha.2020.110543*

*17.	Naghizadeh B, Mansouri MT, Ghorbanzadeh B, Farbood Y, Sarkaki A. Protective effects of oral crocin against intracerebroventricular streptozotocin-induced spatial memory deficit and oxidative stress in rats. Phytomedicine. 2013;20. doi:10.1016/j.phymed.2012.12.019*

*18.	Asalgoo S, Jahromi GP, Hatef B, Sahraei H. The Effect of Saffron Aqueous Extract and Crocin on PTSD Rat Models: The Focus on Learning and Spatial Memory. SSRN Electronic Journal. 2018. doi:10.2139/ssrn.3480403*

*In Figure 2, I cannot see any label of the Y-axes (I guess this should be gram (g)). The graph is not very straightforward and should be presented as body weight increase (Y) per time on X-axes. You can indicate the time of hit 1 and so on. Like this, it looks like the hits lead to a change in body weight. This should be presented as curves, not as bar graphs. I am also confused by the statistics, from the graph it looks to me that there is absolutely no difference e.g. Hit7 TBI vs Saffron TBI. You show a significant change anyways!?*

*Reply: Figure 2 has been redrawn as suggested by the reviewer. The difference in body weight changes is now clearer showing the significant changes after day 9 which corresponds to the 5^th^ hit.*

*You write about the anti-tubulin activity of saffron components. Please explain what you mean! As a biochemist, activity is reserved for enzymes, saffron is not an enzyme. Also what is anti-tubulin activity, does it interfere with polymerization, depolymerization interferes with modification*

*Reply: Yes, the activity is definitely reserved for enzymes, and saffron is not an enzyme as you are specifying. However, Saffron’s main constituent, i.e., safranal, exerts its effects on cytoplasmic components through macromolecular synthesis and cytotoxicity* [19]*. Saffron is classified as an antioxidant* [20] *and anti-inflammatory* [21] *agent with antiproliferative effects that regulates tubulin activity* [22]*. The saffron anti-tubulin activity includes interference in tubulin polymerization where safranal binds to tubulin dimer between the alpha and beta subunit through hydrophobic interactions and one possible hydrogen bond leading to tubulin structural changes* [23]*.*

*Reference:*

19.	Nair SC. Saffron Chemoprevention in Biology and Medicine: A Review. Biotherapy. 1995;10: 257–264.

20.	Nassiri-Asl M, Hosseinzadeh H. The role of saffron and its main components on oxidative stress in neurological diseases: A review. Oxidative Stress and Dietary Antioxidants in Neurological Diseases. Elsevier; 2020. doi:10.1016/B978-0-12-817780-8.00023-2

21.	Fernández-Albarral JA, Ramírez AI, de Hoz R, López-Villarín N, Salobrar-García E, López-Cuenca I, et al. Neuroprotective and Anti-Inflammatory Effects of a Hydrophilic Saffron Extract in a Model of Glaucoma. International Journal of Molecular Sciences. 2019;20. doi:10.3390/ijms20174110

22.	Bie X, Chen Y, Zheng X, Dai H. The role of crocetin in protection following cerebral contusion and in the enhancement of angiogenesis in rats. Fitoterapia. 2011;82. doi:10.1016/j.fitote.2011.06.001

23.	Naghshineh A, Dadras A, Ghalandari B, Riazi GH, Modaresi SMS, Afrasiabi A, et al. Safranal as a novel anti-tubulin binding agent with potential use in cancer therapy: An in vitro study. Chemico-Biological Interactions. 2015;238. doi:10.1016/j.cbi.2015.06.023

*In Figure 3 it is unclear which band was quantified in the NeuN blot, there is a clear double band in the Sham and Saffron Sham but not in TBI. Which band did you use for quantification and how do you know which one is NeuN? Why is it not present in the TBI sample?*

*Reply: The two bands at 48 and 46kDa correspond to the two isoforms of the NeuN/FOX3 protein as described by the antibody manufacturing company EnCor Biotechnology Inc. We quantified the upper band at 48kDa. The overexpression in the TBI group of the 48KDa band hid the lower band.*

*Similarly, in Figure 6C it is unclear which band is SIRT1 and which one was used to quantify. The original blots should be presented without cropping in a supplementary figure.*

*Reply: The original blots were submitted to the journal without any cropping. The two bands represent the SIRT1. The two bands could be for isoforms of SIRT1. We quantified the upper band observed at 120KDa as per the Cell signaling technology datasheet where we purchased the antibody.*

*How do you explain an upregulation of SIRT1 only in TBI brains but not in Saffron sham mice? The effect seems not dependent on Saffron. Please discuss this further.*

*Reply: SIRT 1 is an endogenous neuroprotective factor that confers protection via different pathways. Saffron was observed to enhance SIRT1 gene expression by Abedimanesh et al* [24] *in patients with coronary artery diseases and our study an increase in SIRT-1 was observed protein levels in TBI and a further increase in the saffron-TBI group rather than simple normalization after the injury. The Saffron sham and the sham groups showed similar levels of SIRT-1 implying that saffron does not increase SIRT1 expression. The increase was observed after inducing the injury and thus this increase is associated with the neuroprotective action exerted by saffron.*

*Reference:*

24.	Abedimanesh N, Motlagh B, Abedimanesh S, Bathaie SZ, Separham A, Ostadrahimi A. Effects of crocin and saffron aqueous extract on gene expression of SIRT1, AMPK, LOX1,NF‐κB,and MCP‐1 in patients with coronary artery disease: A randomized placebo‐controlled clinical trial. Phytotherapy Research. 2020;34. doi:10.1002/ptr.6580
